# Supplementary material for: A live yeast supplementation to gestating ewes improves bioactive molecule composition in colostrum with no impact on its bacterial composition and beneficially affects immune status of the offspring
Source: J Nutr Sci. 2022 Feb 7;11:e5. doi: 10.1017/jns.2022.3 (PMC8889232; doi:10.1017/jns.2022.3)
Supplement: Supplementary file 1 [file S2048679022000039sup001.docx]

**Supplementary data**

**Figure S1:** Identification of the differentially expressed compounds achieved by MS/MS. Product ion spectra of commercial standards of 6’acetyl-sialyllatose (6’-ASL) and 6’acetyl-sialyllatosamine (6’-ASLN) show product ion of Neu5Ac at m/z 290.088. The putative, glycolyl containing variants of these compounds within samples had similar MS/MS spectra, with a distinct product ion at m/z 306.0826, indicating that an additional oxygen is present on the sialyl residue, in line with Neu5Gc.

**Figure S2:** Pick area ratio of CMP-Neu5Gc/CMP-Neu5Ac in T5h colostrum from C (green) or SC (red) ewes.

**Figure S3:** OTU PCoA plot according to Bray Curtis distance of T0-colostrum samples from C (red, n = 6) and SC (blue, n = 5) groups.


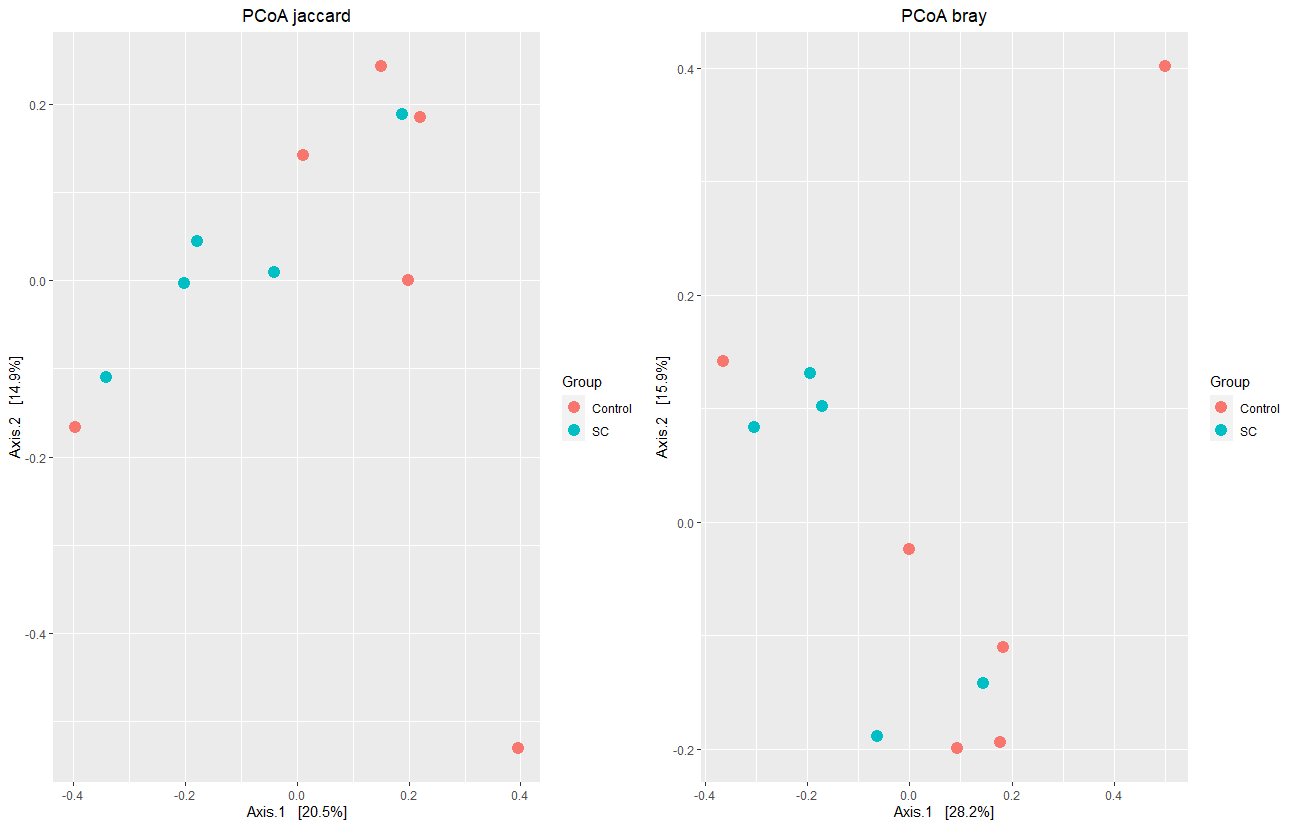


**Table S1**: Composition of the commercial concentrate.

| **Ingredient** | **g/kg of concentrate** |
| --- | --- |
| Corn gluten feed | 179.7 |
| Rapeseed cake | 150.0 |
| Linseed extruded supplement | 146.3 |
| Wheat bran | 127.0 |
| Barley grain | 100.0 |
| Cereal by products | 62.0 |
| Corn meal | 50.0 |
| Sugarcane molasse | 50.0 |
| Beet pulp | 50.0 |
| Wheat grain | 45.0 |
| Supplements (lime carbonate, microminerals) | 40.0 |

**Table S2**: Nutritional composition of the diet offered to the ewes. DM=dry matter; NDF= neutral detergent fiber; ADF= acid detergent fiber.

|  | **Experimental diet** |  |
| --- | --- | --- |
| **Good quality meadow hay** |  |  |
| kg/d/ewe | 2 |  |
| Dry matter | 0.85 |  |
| kg/d/ewe (DM) | 1.7 |  |
| **Concentrate** |  |  |
| kg/d/ewe | 0.8 |  |
| Dry matter | 0.875 |  |
| kg/d/ewe (DM) | 0.7 |  |
| Forage / Concentrate (on DM basis) | 71/29 |  |
| **Daily supply (kg/d/ewe)** |  |  |
| NDF | 1.2693 |  |
| ADF | 0.6817 |  |
| Crude protein | 0.3174 |  |
| Fat (from concentrate) | 0.0351 |  |
| Starch + sugars (from concentrate) | 0.2156 |  |

**Table S3:** Targeted analytes quantified by LC-HRMS.

| **Abbreviation** | **Analyte** | **Formula** | **[M-H]^-^** | **RT (min)** |
| --- | --- | --- | --- | --- |
| 3’-ASL | 3'-N-acetylneuraminyl-lactose | C_23_H_39_NO_19_ | 632.2021 | 4.42 |
| 3’-GSL | 3'-N-glycolneuraminyl-lactose | C_23_H_39_NO_20_ | 648.1964 | 4.64 |
| 6’-ASL | 6'-N-acetylneuraminyl-lactose | C_23_H_39_NO_19_ | 632.2021 | 4.62 |
| 6’-GSL | 6'-N-glycolneuraminyl-lactose | C_23_H_39_NO_20_ | 648.1964 | 4.80 |
| 3’-ASLN | 3'-N-acetylneuraminyl-N-acetyllactosamine | C_25_H_42_N_2_O_19_ | 673.2285 | 4.25 |
| 3’-GSLN | 3'-N-glycolneuraminyl-N-acetyllactosamine | C_25_H_42_N_2_O_20_ | 689.2233 | 4.45 |
| 6’-ASLN | 6'-N-Acetylneuraminyl-N-acetyllactosamine | C_25_H_42_N_2_O_19_ | 673.2285 | 4.37 |
| 6’-GSLN | 6'-N-glycolneuraminyl-N-acetyllactosamine | C_25_H_42_N_2_O_20_ | 689.2233 | 4.56 |

**Table S4:** Characteristics (m/z, retention time (RT) and putative ID) of differentially expressed features from positive and negative ionization modes, at 0, 5 and 72 h after lambing at p-value < 0.01, according to t-test between SC and C groups.

|  |  |  |  | **p-values** | | |
| --- | --- | --- | --- | --- | --- | --- |
| **ionization mode** | **m/z** | **RT (min)** | **putative ID** | **0h** | **5h** | **72 h** |
| positive | 157,17009 | 4,923592 | C_9_H_2_0N_2_ |  | 0,000509 |  |
| positive | 167,01244 | 11,3731 |  |  | 0,005154 |  |
| positive | 189,13474 | 4,897401 | homo-Arg | 0,002974 |  |  |
| positive | 204,12311 | 3,197402 | acetylcarnitine |  | 0,000311 |  |
| positive | 258,11045 | 5,674051 | Glycerophosphocholine |  | 0,003336 |  |
| positive | 263,16775 | 1,897495 |  | 0,008535 |  |  |
| positive | 284,33096 | 11,29636 | noise |  | 0,002655 |  |
| positive | 290,08908 | 0,720561 |  |  | 0,000254 |  |
| positive | 292,20126 | 0,642597 |  | 0,007958 | 0,002638 |  |
| positive | 308,09742 | 4,611503 |  |  | 0,003651 |  |
| positive | 311,0774 | 3,67844 |  |  |  | 0,002172 |
| positive | 364,12214 | 3,714425 |  |  | 0,007138 |  |
| positive | 376,25871 | 0,616248 |  |  | 0,009907 |  |
| positive | 381,07872 | 3,629511 |  |  |  | 0,001469 |
| positive | 381,07906 | 3,976418 |  |  | 0,00542 |  |
| positive | 382,08188 | 3,396364 |  |  |  | 0,001992 |
| positive | 393,28529 | 0,616248 |  |  | 0,007706 |  |
| positive | 395,29085 | 0,61621 |  | 0,008622 |  |  |
| positive | 421,31634 | 0,616685 |  |  | 0,001275 |  |
| positive | 422,31968 | 0,61716 |  |  | 0,008526 |  |
| positive | 432,23713 | 0,627395 |  |  | 0,000612 |  |
| positive | 449,34751 | 0,615718 |  | 0,002977 | 0,009812 |  |
| positive | 525,13056 | 4,087246 |  | 0,005269 |  |  |
| positive | 691,23962 | 4,560937 | SLN_O | 0,004065 | 0,003 |  |
| positive | 692,24285 | 4,556318 | SLN_O | 0,004142 | 0,003019 |  |
| positive | 713,22131 | 4,561031 | SLN_O | 0,002521 | 0,001055 |  |
| positive | 714,22439 | 4,560253 | SLN_O | 0,002953 | 0,00087 |  |
| positive | 715,22636 | 4,556318 | SLN_O | 0,00335 | 0,001921 |  |
| positive | 814,24268 | 4,366603 |  | 0,006686 |  |  |
| negative | 178,81327 | 1,392473 |  | 0,002988 |  |  |
| negative | 297,73256 | 2,043758 |  | 0,000823 |  |  |
| negative | 316,73821 | 0,891627 | Copper diIodine |  | 0,001001 |  |
| negative | 318,73637 | 0,891945 | Copper diIodine |  | 0,000815 |  |
| negative | 322,04392 | 4,270469 |  |  |  | 0,009128 |
| negative | 332,0833 | 2,947639 |  |  | 0,007637 |  |
| negative | 332,08335 | 2,410796 |  |  |  | 0,004293 |
| negative | 332,08338 | 0,399051 |  | 0,000245 |  |  |
| negative | 332,08365 | 1,344488 |  | 0,004243 |  |  |
| negative | 629,05825 | 4,652889 |  |  |  | 0,008088 |
| negative | 648,19769 | 4,623225 | 3’SL+O |  | 0,000774 |  |
| negative | 666,14627 | 3,509148 |  |  |  | 0,004436 |
| negative | 689,22418 | 4,564851 | 6’SLN+O | 0,00242 | 0,002257 |  |
| negative | 690,22785 | 4,557011 | 13C 6SLNO | 0,001758 | 0,003294 |  |
| negative | 955,29114 | 5,12 | DSL_2_O |  | 0,007588 |  |
